# Using YouTube to Disseminate Effective Vaccination Pain Treatment for Babies

**DOI:** 10.1371/journal.pone.0164123

**Published:** 2016-10-03

**Authors:** Denise Harrison, Jodi Wilding, Amanda Bowman, Ann Fuller, Stuart G. Nicholls, Catherine M. Pound, Jessica Reszel, Margaret Sampson

**Affiliations:** 1 Children’s Hospital of Eastern Ontario (CHEO) and CHEO Research Institute, Ottawa, Ontario, Canada; 2 School of Nursing, University of Ottawa, Ottawa, Ontario, Canada; National Eye Institute, UNITED STATES

## Abstract

**Background:**

Infant vaccinations are necessary for public health, but are painful, causing distress to the infant and caregivers. Breastfeeding and sucrose effectively reduce infants’ pain during vaccinations, and these strategies are recommended in health care provider (HCP)-targeted education and vaccination pain guidelines. However studies show these strategies are infrequently used. YouTube is a popular medium to publicly share and watch videos, and many consumer posted YouTube videos show distressed infants being vaccinated with no pain treatment. The aims of this study were to evaluate the reach and impact of a consumer-targeted YouTube video demonstrating use of effective pain reduction strategies during infant vaccinations.

**Methods:**

A brief consumer-targeted video showing two infants being vaccinated was posted onto YouTube on October 2013. One infant was breastfed and another infant received sucrose by mouth before and during the injection. A link to a viewer survey was visible on a banner near the end of the video. An intensive strategically planned knowledge dissemination strategy using the media, social media and messages to professional organizations took place to promote the video. Data analysis of the viewer survey, YouTube analytics of the reach of the video in terms of number of views, country of viewers, and comments relating to the video took place 12 months after the video was posted.

**Results:**

Twelve months after posting, the video had 65,478views, 68 comments, 245 likes, 17 dislikes, and 90 shares. Average duration of viewer time was 65% of the video. The viewer survey was completed by 156 (0.24%) viewers; 90 (58%) answered as HCPs and 66 (42%) as parents. Survey results showed that the video was persuasive; intent to use or support breastfeeding or sucrose was high in both parents and HCPs after viewing the video. Comments posted were often emotional in nature, and were related to anti-vaccination (n = 26, 38%); effectiveness or positive personal experiences (n = 21, 32%); research team comments or promotion (n = 12, 18%); pro-vaccination (n = 6, 8%) and barriers to using breastfeeding or sucrose during vaccinations (n = 3, 4%).

**Conclusion:**

The video posted onto YouTube demonstrating effective pain treatment during infant vaccinations was viewed by large numbers of people around the world, however the response rate to the linked survey was extremely low. Using YouTube videos for knowledge dissemination has an extensive reach, however it is difficult to evaluate impact on behaviours and practices.

## Introduction

Early childhood vaccinations are vital for public health.[1] However, vaccinations are painful and cause distress in infants,[2,3] which can negatively affect parents and health care providers (HCPs)[4] as well as lead to a fear of needles, parental non-adherence to immunization schedules and avoidance of medical care.[5] Considerable evidence exists of analgesic effects of breastfeeding and sweet tasting solutions for infants during vaccinations,[6–10] which, if used consistently during early childhood vaccinations, may reduce the incidence and severity of the aforementioned pain-related outcomes. Professional guidelines recommend using sucrose and breastfeeding,[7,11,12] or front-to-front holding,[5,7] however a number of reports highlight a gap between knowledge and clinical practice.[13–16] This knowledge to practice gap behooves HCPs and health care researchers to work at improving the translation of knowledge into action.

Approaches to improving the uptake of research evidence has traditionally focused on HCPs, [17] however more recently, attention is being focused on the potential utility of consumer-oriented health information.[18–20] In addition, the internet and social media is now used extensively for communication, and sites such as YouTube are frequently used by consumers to search for health information.[21,22] However, as so much health information is sought online, it is important that the available information is accurate, evidence-based, and practices portrayed are in line with best evidence. Yet research shows that this is not always the case, and posted health information can be incorrect and misleading.[23–26] For example, in a content analysis of 153 YouTube videos on immunization, one quarter concerned childhood vaccination.[26] Of these, the majority included negative messaging about pain (15 videos), and adverse events (16 videos), such as autism or permanent injury. Publicly available negative messages highlighting the pain of early childhood vaccination, but yet offering no treatment options to reduce pain, and the risk of adverse events are not in line with positive public health messages. In addition, a systematic review of 142 YouTube videos showing infant vaccinations portrayed highly distressed infants and found that there was no use of breastfeeding or sucrose for pain treatment during the vaccinations.[2] Given the potential utility of online consumer-oriented health information but the concomitant negative messaging about pain management during vaccination, there is a need to develop and disseminate high quality evidence-based information regarding pain management techniques during vaccinations in infants and children.

A consumer-targeted educational video demonstrating the use of effective, recommended vaccination pain treatment strategies was posted on YouTube, and the reach and impact of the video were evaluated

## Materials and Methods

Our study team produced a brief parent-targeted video showing two infants being vaccinated while i) being breastfed; ii) receiving sucrose by mouth before and during vaccinations and posted the video onto YouTube (tinyurl.com/BSweet2Babies). The video was filmed by the research team’s affiliated hospital audiovisual team, in a children’s hospital outpatient clinic. The brief (1 minute 35 seconds) video included simple captions in Canada’s two official languages (English and French) and a brief written statement of the effectiveness of breastfeeding and sucrose as pain reduction strategies during infant vaccinations. More detailed information on how parents and HCPs can implement breastfeeding and sucrose during infant vaccinations was provided in the description accompanying the video.

The video contained a link to a REDCap (Research Electronic Data Capture) online survey[27]. The survey included ten closed-ended questions asking viewers if they were health care providers or parents; if they had used breastfeeding or sucrose previously for pain reduction; if they intended to use breastfeeding or sucrose during vaccination after viewing the video; and how the viewer found the video. The questions were piloted with study team members for clarity and content and revised accordingly. The link to the viewer survey appeared in an annotation banner near the end of the video. After six months of posting the video onto YouTube (April 2014), it was ascertained that the response rate to the linked survey was low, and the average view time of the video was only 1 minute 5 seconds. In an attempt to address the low survey response rate, the link to the online survey was moved earlier in the video at 50 seconds to facilitate viewers seeing the banner with the survey link before they stopped their viewing of the video.

### Dissemination of the YouTube video

In collaboration with the team’s affiliated public relations and communication teams, a comprehensive video dissemination plan was put into place to maximize views during the intervention period. This included the following activities.

The video was posted on YouTube on October 21, 2013. Data was collected for 12 months following the posting of the video, from October 21, 2013 to October 20, 2014. The dissemination plan was implemented as follows.

1. The posting of the video and dissemination plan coincided with the annual national and regional flu vaccination promotion efforts.
2. Press releases were sent to media resulting in 18 media interviews including: two radio interviews, seven television interviews and nine text publications (online and print) in Canada, United States of America (USA) and Australia.
3. Pitch letters were sent to popular parent blogger groups (e.g., The Yummy Mummy’s Breastfeeding Blog, Parenting.com, The Bump.com), and professional organizations or mailing lists (e.g. Pediatric-Pain List, Nurses Who Vaccinate.blogspot)
4. Key words were used to maximize the chances of the intervention video being retrieved in YouTube searches. For example, “baby” and “vaccination” were included in the title and their synonyms (i.e. infant, immunization) were used throughout the description of the video. In addition, tags (key words) were used to capture possible search terms that viewers would use to find information about vaccination pain. For example: Baby vaccination; Baby injection; Flu shot; Vaccination; Vaccines; Breastfeeding; Pain.
5. Team members’ personal and affiliated organizational social media sites (YouTube, Facebook, Twitter) were used to post and promote the video.
6. Posters and pocket sized cards with information about the video (title, URL, barcode to scan with mobile device to access the video) were disseminated at 31 medical clinics in the inner city of Ottawa, and shared at six national and international professional research conferences.
7. The following comment was posted onto all other YouTube videos identified showing infant vaccinations: “Research shows that breastfeeding and/or small amounts of sugar water effectively reduce pain during vaccinations! Watch this video: Baby vaccination; the secret to a calm and peaceful immunization!”

Data analysis of the viewer survey and YouTube analytics of the reach of the video in terms of number of views, country of viewers and comments relating to the video took place 12 months after the video was posted. Results of the reach of the video and viewer survey were summarized using descriptive statistics using means and their standard deviations (SD) if data was normally distributed, and medians and interquartile ranges (IQR) if data was skewed. Comments were reviewed by three authors (DH, JW and JR) and organized by consensus into categories. Direct quotes are used to illustrate the identified categories.

### Ethics

The parents and nurses portrayed in the videos provided written informed consent to have the video files posted onto YouTube. The consent processes, as well as the posted intervention video and the study were approved by the first author’s affiliated hospital research ethics board.

## Results

The infant vaccination YouTube video view count was 65,478 during the 12-month period following posting (S1 Fig). The average view duration was 1 minute 2 second or 65% of the video. The video was viewed in 175 countries, with the top five viewing countries being USA (24%), Canada (16%), Saudi Arabia (6%), United Kingdom (4%), and India (4%). Between the YouTube video page and the team’s affiliated hospital Facebook page, the video received a total of 69 comments, 245 likes, 17 dislikes, and 90 shares. One comment was removed from the YouTube page as it was sexually explicit in nature and did not comply with the hospital’s social media policy. Of the 68 comments that were analyzed, 26 (38%) were categorized as anti-vaccinations; 21 (32%) as positive personal experiences or the effectiveness of the pain reduction strategies; 12 (18%) were research team comments or promotion; six (8%) were pro-vaccination and there were three comments (4%) related to barriers to using breastfeeding or sucrose during vaccinations. Table 1 provides a sample of viewers’ comments about the video.

**Table 1:** Viewer comments.

Comment	N (%)	Examples
**Anti-vaccination**	26 (38)	• How to comfort your baby before poisoning them • Don’t vaccinate him you idiots • Do you know what is better? NOT TO VACCINATE!!! poor baby!!! • Your baby got mercury and aluminum from the vaccines. (3 responses in this conversation) • Wow, I hope mommy reads the inserts of all the jabs she is going to get him • Mercury, aluminum is wonderful for babies. Do your research. • Vaccines should not be given to babies. Sad • Don’t get one that is most peaceful of all. • Don't vaccine…No ouch and no problems for baby..pretty simple
**Effectiveness or positive personal experiences**	21 (32)	• No one wants to see a crying baby! The techniques used in the video are good for the baby, parent and nurse! It’s a win, win situation!! • So good. Especially to use breastfeeding • I've always breastfed AFTER my babies had their needles, but now I'm going to start BEFORE they have it. • So simple and low tech. applicable in resource limited settings as well. • I wish I had known about it when I was breastfeeding. But I did try the sucrose when my daughter was an older infant, and it did work. • I breastfed my daughter when she received needles as a baby. It not only helped her, it comforted me to know I could help • I always nursed my girls during shots-it was a huge help • I have nursed all 4 of my kids for almost every needle. When we had a new family doctor (new as in fresh out of school), she couldn't believe how calm my twins were because of it. She said, "I'm going to tell all my mom’s to nurse during vaccines!" • The nurses gave my son sugar water just before and during blood work when he was in the NICU. It seemed to help him with the pain. I breastfed my daughter when she was a baby during vaccinations and it worked everytime! • At our family doc today for immunizations and talked about the video. She has always recommended nursing as it helps keep them calm. It was a quiet cry free first round of immunizations today!
**Pro Vaccine**	6 (8)	• You realize that there is no proof that vaccines do anything but help children like this? Sure, some rare individuals may be allergic to an ingredient (i.e. eggs) but complications are exceedingly rare. Vaccines save lives. Anti-vaccine idiots do nothing but spread FUD and weaken herd immunity against common illnesses, harming everyone, INCLUDING YOUR CHILDREN! Anti-vaccine morons kill more children in a year than vaccines have since their invention! • Maybe if some of had seen children suffer and sometimes die from these preventable diseases you might feel differently. That's the really sad part. • Only one of the single use vial influenza vaccines on the market in Canada contains thimerosol. And thimerosol has not been shown to cause neurological problems of autism. But I appreciate your sharing your comments because it gives us a chance to address some of myths associated with vaccines.
**Barriers to Use**	3 (4)	• Wow! How many times I asked nurses, for let me breast feed my son trough vaccination and they said NO! Because "is uncomfortable for me to do my job", they said. • Thank you CHEO for putting this out there. I tried to nurse during my 2 month shot and was told no by the nurse. Hopefully this video will help change her mind. • Surprising, I knew this yet a nurse in the NICU at [hospital] refused to let me breastfed while taking blood sample from my newborn because she said the baby associate the pain with nursing then will not nurse well. My baby was nursing well and still is. We have to go for more blood work soon, I’ll make sure to help my child cope well with the pain this time.
**CHEO Research team and public relations department comments or plugs**	12 (18)	• Parents- ask your Doctors and Nurses about breastfeeding or sugar water and best way to hold your baby, to help reduce your babies’ pain during vaccinations. Be Sweet to Babies! • Simple, free or very cheap, and importantly—effective!!

The online survey linked to the video was completed and submitted by 156 viewers (S1 Table). Based on the number of views (65,478), this was a response rate of only 0.24%. Over half of the respondents were healthcare providers (n = 90, 58%) and 66 (42%) were parents. The most common ways survey respondents found the video were through HCPs’ recommendations (n = 70, 43%), Facebook, Twitter or e-mail (n = 30, 19%) or family and friends (n = 25, 15%).

As presented in Table 2, 25 parent respondents (38%) had breastfed their infants during vaccinations. After seeing the video, 56 (86%) parents answered they would breastfeed during their infant’s subsequent immunizations. Only nine (14%) parents had used sweet solutions previously, however after seeing the video, 47 (73%) answered they would use sweet solutions during their infant’s subsequent immunizations. Of the HCPs respondents, 53 (60%) had previously encouraged mothers to breastfeed during vaccination, and following viewing the video, 85 (97%) reported they would encourage mothers to breastfeed during vaccination. Thirty-nine (44%) HCPs routinely gave sucrose to infants, and following viewing the video, 82 (94%) reported they would use sucrose during subsequent infant vaccinations (n = 82, 94%) (Table 2).

**Table 2:** Viewer survey results.

Respondent	Survey question	YESN (%)
**Parents**	Have you breastfed your baby during immunization? (n = 65)	25 (38)
**(n = 66)**	After seeing video, would you use breastfeed during immunization?(n = 65)	56 (86)
	Have you used sweet solutions during immunization? (n = 65)	9 (14)
	After seeing video, would you use sweet solutions forimmunizations? (n = 64)	47 (73)
**Health care providers**	Do you encourage mothers to breastfeed during immunization?(n = 89)	53 (60)
**(n = 90)**	After seeing this video, would you encourage mothers to breastfeed during immunization? (n = 88)	85 (97)
	Do you routinely give sucrose to infants for immunization? (n = 88)	39 (44)
	After seeing this video, would you use sucrose for immunizations?(n = 87)	82 (94)

NB: Not all respondents answered each section of the survey. The number (N) of respondents for each question is stated.

## Discussion

This study showed that a brief consumer (parent)-targeted educational video disseminated by the YouTube social media platform and demonstrating effective pain reduction (breastfeeding and sucrose) during infant vaccination attracted large numbers of viewers from around the world. This approach resulted in wider viewership than is typical for health-related YouTube videos. [2,28–30] For example, the median number of views for concussion-related videos included in a systematic review of 100 YouTube videos, was just over 26,000 [30] and a systematic review of 47 YouTube videos of first-aid for burn treatment, reported an average of 6742 views.[29] The large number of views for our infant pain vaccination video may be due to many factors, including our strategically planned extensive dissemination strategies, including media engagement, video dissemination through the investigators’ and their organizations’ social media platforms of Facebook and twitter, as well as the targeting of professional and consumer groups and their respective blogs, newsletters or mailing lists. In addition, the topic of infant vaccination received extensive media coverage during our study period, including on controversial subjects such as measles outbreaks (http://www.cbc.ca/news/canada/edmonton/measles-vaccines-should-they-be-mandatory-1.2629890) and stories refuting the link between vaccinations and autism (http://www.dailymail.co.uk/news/article-2632526/There-NO-link-autism-childhood-vaccines-major-new-survey-found.html). Stories such as these may have influenced the number of people searching the internet for childhood vaccine related information, further underscoring the importance of ensuring that information concerning effective pain treatment during vaccination has an online presence, is easy to find, and is targeted appropriately at the average online viewer.

The YouTube video posted for this study is just one of a number of YouTube videos relating to pain reduction during childhood vaccination posted by health care researchers or providers,[31,32] and is indicative of rapidly increasing practices by health care researchers and providers, health agencies and health product companies of posting consumer targeted videos onto YouTube. An example is the Help Eliminate Pain in KIDS (HELPinKIDS) team videos ‘Managing Infant Pain—Helpful tips’, an 8-minute educational video posted onto YouTube in November 2012 (https://www.youtube.com/watch?v=jxnDc2PxGUc&list=PLJH3y0duq2ZEQ_KkfKVkcLwZUk3HPV6xj&index=1) and an updated version, a 13-minute educational video, “Reduce the Pain of Vaccination in Babies’ posted onto YouTube in March 2015 (https://youtu.be/5Oqa1Fag5eQ). However, in the first 12 months of posting, the original video had less than 5000 views, and the updated video has had just over 5000 views after 10-months of posting. These number of views are relatively small compared to many of the consumer-posted videos showing distressed crying babies receiving injections.[2]

For the 156 viewers who completed the viewer survey linked from our video, results indicated the video was persuasive. Almost 100% of HCPs indicated they would support use of breastfeeding or sucrose for future infant vaccinations, 86% of parents responded they would breastfeed and 73% of parents would use sucrose during future vaccinations. These findings of high intent to use or encourage effective pain treatment strategies are promising. However methods to effectively evaluate the *impact* of health education videos posted onto YouTube are to date lacking. We cannot determine if the high *intent* to use or encourage breastfeeding or sucrose during vaccination, as demonstrated in our survey results, actually results in use of these strategies during future vaccinations. In addition, the extremely low response rate of the linked survey to our video; less than 1% of video viewers, is a limitation to using data from surveys linked to YouTube and suggests that this method of data collection may have limited use as an approach to evaluate the impact on consumer behaviours, parent advocacy, beliefs or on clinical practices. This finding corroborates findings of a survey regarding the use and beliefs of using social media for health research, completed by 181 faculty members at the Johns Hopkins School of Public Health, United States of America.[33] Perceptions were that using social media, including YouTube, was important to disseminate knowledge but less useful to do research, or to obtain information. Our results are also consistent with recently published work by Farkas et al, who reviewed YouTube videos focusing on pediatric needle pain treatment.[32] Their results indicated that while the videos were deemed to be of high quality, evaluation is needed to determine whether the use of social media as an education tool meets the needs of target audiences, and can improve management of pediatric needle pain.[32]

Another factor to consider is that utilizing social media to deliver health-related information, means that there is no control over the fidelity of the intervention (the information being delivered). For example, despite our study video being only 1 minute 35 seconds in duration, well within the average length of the most popular viewed YouTube videos of 2 minutes 54 seconds,[34] the average viewing time was just over 1 minute; only two-thirds of the video. This highlights the limitation of relying on health-related messages actually being delivered as planned to viewers. The short view duration on average also highlights the need for future videos posted onto YouTube targeted at consumers to be extremely short with engaging key messages.

Although the aim of our consumer (parents of infants)-targeted video was to demonstrate use and effectiveness of breastfeeding and sucrose for pain reduction during vaccinations, 40% of the comments posted were anti-vaccination and often highly emotional in nature, which in turn, promoted replies or counter-arguments of a pro-vaccination nature. The use of the internet as a public forum for expressing anti-vaccination messages including messages about dangers of vaccines, has been previously described.[35] This highlights that using such a public forum to disseminate research with the aim of improving practices can result in unintended uses, which HCPs and researchers need to be cognisant of.

### Strengths and Limitations

Key strengths of this study relate to the unique and innovative nature of the study methods, bringing a new dimension to health research using social media to disseminate knowledge. Key limitations include extremely low response rate to the linked online survey, and the lack of ability to draw associations between the YouTube video and clinical outcomes.

### Recommendations for future research and practice

Using social media to disseminate knowledge has the potential to reach a large audience, making this a promising knowledge dissemination strategy. However, evaluation of effectiveness of the knowledge being disseminated is currently limited. To rigorously evaluate the effectiveness of YouTube videos on changing practices, traditional randomized controlled trials (RCT) or cluster RCT research methods, conducted alongside knowledge dissemination strategies using social media platforms may be required.

## Conclusion

Our brief consumer targeted educational YouTube video demonstrating effective pain reduction during infant vaccination had a large reach, as evidenced by more than 68,000 views in the first 12 months of posting. However, the response rate to the viewer survey was extremely low. This suggests that surveys linked from YouTube videos may not be a useful method of data collection. This highlights the limitations of evaluating effectiveness of publicly accessible health messages posted on YouTube and evaluating their impact on practice change. Further studies using social media platforms for knowledge dissemination are needed to determine the best methods of studying impact. Such effectiveness of knowledge translation intervention studies may need to be conducted alongside traditional and tightly controlled RCTs.

## Supporting Information

S1 Fig YouTube Analytics. Data used from YouTube Analytics after video had been posted for 12 months.
(PDF)

S1 Table Survey Responses. Raw survey data from parents and health care providers.
(XLSX)
