# Supplementary material for: Using YouTube to Disseminate Effective Vaccination Pain Treatment for Babies
Source: PLoS One. 2016 Oct 3;11(10):e0164123. doi: 10.1371/journal.pone.0164123 (PMC5047634; doi:10.1371/journal.pone.0164123)
Supplement: S1 Fig — Data used from YouTube Analytics after video had been posted for 12 months. (PDF) [file pone.0164123.s001.pdf]

CREATOR STUDIO

DASHBOARD

VIDEO MANAGER

LIVE STREAMING

COMMUNITY

CHANNEL

ANALYTICS

Overview

Realtime

Revenue reports

Revenue

Ad rates

Watch time reports

Watch time

Audience retention

Demographics

Playback locations

Traffic sources

Devices

Engagement reports

Subscribers

Likes and dislikes

Videos in playlists

Comments

Sharing

Annotations

Cards

CREATE

Help and feedback

The demographics report has had a makeover to allow deeper insights. Learn more

Overview

Comparison... Groups Export report

Baby vaccination; the secret to a calm and peac

Search for locations

First 365 days

Subscribed & not subscribed

All YouTube products

CHEOvideos ›

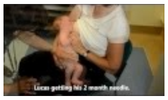

Baby vaccination; the secret to a calm and peaceful immunization

Created: Oct 21, 2013 • Duration: 1:36 • Privacy setting: Public

VIDEO

First 365 days (Oct 21, 2013 – Oct 20, 2014)

Watch time

Minutes

67,862

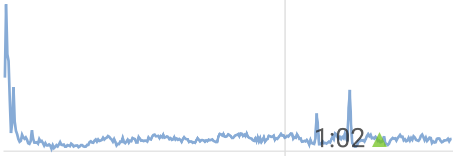

Average view duration

Minutes

1:02

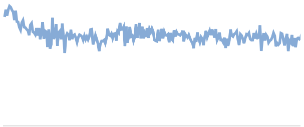

Views

65,478

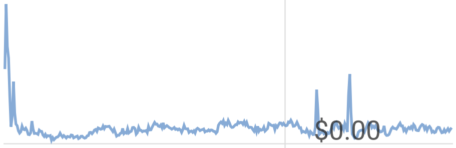

Your estimated revenue *

$0.00

Likes	Dislikes	Comments	Shares *	Videos in playlists *	Subscribers
82	17	42	65	40	11

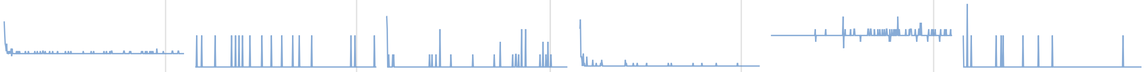

Top geographies

Watch time

- United States (24%)
- Canada (16%)
- Saudi Arabia (6.4%)
- United Kingdom (4.2%)
- India (3.7%)

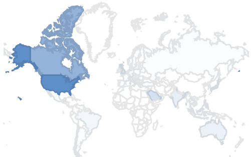

Gender

Views

- Male (43%)
- Female (57%)

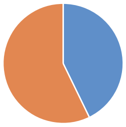

Traffic sources

Watch time

- Suggested videos (37%)
- Direct or unknown (25%)
- YouTube search (19%)
- Other (19%)

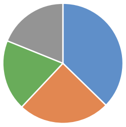

Playback locations

Watch time

- YouTube watch page (88%)
- Embedded in external websites and apps (12%)
- YouTube other (0.2%)
- Other (0.0%)

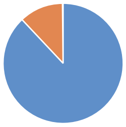

Skip navigation

CA

Search

Upload

5

About Press Copyright Creators Advertise Developers +YouTube

Terms Privacy Policy & Safety Send feedback Try something new!
